# Towards a quantitative determination of strain in Bragg Coherent X-ray Diffraction Imaging: artefacts and sign convention in reconstructions

**DOI:** 10.1038/s41598-019-53774-2

**Published:** 2019-11-22

**Authors:** Jérôme Carnis, Lu Gao, Stéphane Labat, Young Yong Kim, Jan P. Hofmann, Steven J. Leake, Tobias U. Schülli, Emiel J. M. Hensen, Olivier Thomas, Marie-Ingrid Richard

**Affiliations:** 1 Aix Marseille Université, CNRS, Université de Toulon, IM2NP UMR 7334, 13397 Marseille, France; 2 ID01/ESRF, The European Synchrotron, 71 Avenue des Martyrs, 38000 Grenoble, France; 3 Laboratory for Inorganic Materials and Catalysis, Department of Chemical Engineering and Chemistry, P. O. Box 513, 5600 MB Eindhoven, The Netherlands; 4 Deutsches Elektronen-Synchrotron (DESY), D-22607 Hamburg, Germany

**Keywords:** Imaging techniques, Nanoparticles

## Abstract

Bragg coherent X-ray diffraction imaging (BCDI) has emerged as a powerful technique to image the local displacement field and strain in nanocrystals, in three dimensions with nanometric spatial resolution. However, BCDI relies on both dataset collection and phase retrieval algorithms that can induce artefacts in the reconstruction. Phase retrieval algorithms are based on the fast Fourier transform (FFT). We demonstrate how to calculate the displacement field inside a nanocrystal from its reconstructed phase depending on the mathematical convention used for the FFT. We use numerical simulations to quantify the influence of experimentally unavoidable detector deficiencies such as blind areas or limited dynamic range as well as post-processing filtering on the reconstruction. We also propose a criterion for the isosurface determination of the object, based on the histogram of the reconstructed modulus. Finally, we study the capability of the phasing algorithm to quantitatively retrieve the surface strain (*i.e*., the strain of the surface voxels). This work emphasizes many aspects that have been neglected so far in BCDI, which need to be understood for a quantitative analysis of displacement and strain based on this technique. It concludes with the optimization of experimental parameters to improve throughput and to establish BCDI as a reliable 3D nano-imaging technique.

## Introduction

Understanding the role of atomic displacement in determining physical properties and chemical reactivity of nanocrystals requires quantitative characterisation of the strain field with a good spatial resolution in 3D (≤10 nm) and a high strain sensitivity (<10^−3^) in the material being probed. Bragg Coherent X-ray Diffraction Imaging (BCDI) fulfills these requirements. The technique appeared in the 2000s^1,2^ and is finding application in *in situ* and *operando* studies^3–5^. This lens-less technique is based on inverse microscopy and employs digital methods to replace X-ray imaging lenses. Illuminating an isolated object by an X-ray beam with coherence lengths larger than the object, and measuring its oversampled 3D diffraction pattern, it is possible to reconstruct a complex amplitude object using iterative algorithms^6^. In Bragg geometry, the reconstructed phase is related to the projection of the displacement field, $\overrightarrow{u}(\overrightarrow{r})$, along the measured wave vector transfer $\overrightarrow{q}\,$ ^7^. Taking the gradient of this displacement allows direct extraction of one of the corresponding component of the strain tensor with the high sensitivity common to X-ray diffraction measurements^8^.

Often, a qualitative understanding of the strain and displacement components is possible, but since the technique is extending towards *in situ* and *operando* studies, it is of primary importance to get quantitative information and to probe subtle displacement and strain variations. For example, the concept of strain engineering appeared recently in catalysis, where the goal is to tune the surface strain of a catalyst to improve its activity and/or selectivity in a particular chemical reaction^9,10^. Understanding the impact of artefacts induced by both dataset collection and phase retrieval algorithms (*i.e*., for instance, aliasing, detector gaps, detector dynamic range, *etc*.) on the reconstructed strain is therefore an important milestone for the credibility of the technique when applied to more complex environments. Methodical investigation of the performance and capability of phase retrieval has been approached with numerical simulations^11–19^. For instance, it has been demonstrated^11^ that a minimum dynamic range of 10^6^ is needed for the intensity measurement to reach ultimate reconstruction performance and that the best resolution can be achieved with an overall oversampling ratio beyond 30. However, there is no tool to understand and distinguish the phase components caused by the real lattice displacement from the phase fluctuations that are statistical uncertainties introduced by the phase retrieval process itself. These artefacts affect the accuracy and reliability of the recovered modulus and phase and cannot be associated to physical phenomena in the measured crystal.

First, we discuss the formal mathematical relation between the retrieved phase and displacement field. The displacement and the retrieved phase may have an opposite sign depending on the mathematical convention of the fast Fourier transform (FFT) used in the phasing algorithm. The relation between the retrieved phase and displacement has been evoked in ref.^20^, and we use here a simple model to demonstrate it. Then, we use numerical simulations on a realistic system with full control of measurement conditions to quantify the phase fluctuations: detector size, detector gap width and position relative to the Bragg peak, dynamic range, presence or not of noise. We also probe the effect of applying a filter after phase retrieval on the reconstruction quality. Finally, we propose a criterion for isosurface determination, based on the histogram of the reconstructed modulus. We study the capability of the phasing algorithm to retrieve quantitatively the strain of the surface voxels, which is an important parameter in surface physics and chemistry of materials. The study proposes optimum experimental conditions and tools to achieve optimum BCDI.

## Results

### Mathematical formalism between retrieved phase and displacement field

**Figure 1:**
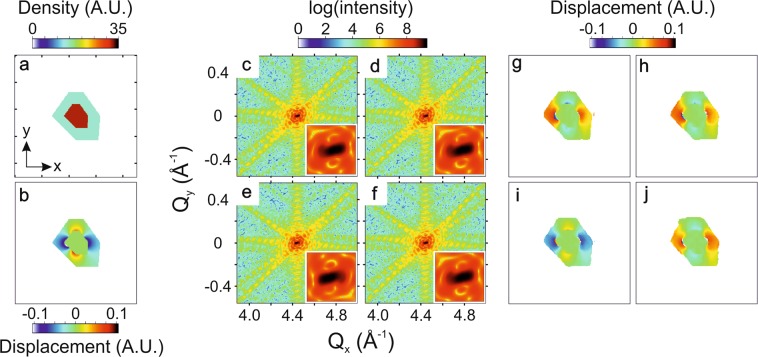
(**a**) Density (tick spacing corresponds to 25 nm) and (**b**) displacement u_x_ of the model used for the simulation. The displacement u_y_ is fixed to zero. (**c**) Kinematic sum with positive convention. (**d**) Kinematic sum with negative convention. This gives the same diffraction pattern as the positive convention. (**e**) FFT of the complex object calculated using Python. (**f**) FFT of the complex object with displacement field of opposite sign, calculated using Python. A zoom on the center of the Bragg peak is shown at the bottom right corner. From this we conclude that the correct and unique diffraction pattern corresponding to our complex object is the one in (**f**) for FFT calculations. (**g**–**j**) show the displacement retrieved from diffraction patterns in (**c**–**f**) respectively using the Python-based phasing algorithm.

Labat *et al*. mentioned briefly that the retrieved phase and the retrieved displacement are of opposite sign as a result of the FFT convention used by their Python-based phasing algorithms^20^. This is a critical point if one wants to perform a quantitative analysis of the displacement field and strain to distinguish compressive or tensile regions in a nanocrystal. We believe that it deserves a more in-depth description. Starting with a 2D model with a known real-space orientation, electron density and displacement u_x_ (u_y_ = 0, see Fig. 1(a,b), we calculate the diffraction pattern using a Python-based FFT algorithm and the kinematical sum (sum over all nodes/atoms). The kinematical sum gives a unique diffraction pattern of intensity I(*q*_*x*_, *q*_*y*_) of the complex-valued real-space object, whatever the (positive or negative) convention used for the phase term: $\tilde{\Omega }(x,y)=\rho (x,y){e}^{\pm i[{q}_{x}.(x+{u}_{x})+{q}_{y}.y]}$. Figure 1(c,d) correspond to diffraction patterns of intensity ${\rm{I}}({q}_{x},{q}_{y})={\Vert \sum _{x,y}\rho (x,y){e}^{+i[{q}_{x}.(x+{u}_{x})+{q}_{y}.y]}\Vert }^{2}$ and ${\Vert \sum _{x,y}\rho (x,y){e}^{-i[{q}_{x}.(x+{u}_{x})+{q}_{y}.y]}\Vert }^{2}$, respectively, which are identical. When we calculate the FFT of the complex object $\tilde{\Omega }(x,y)\,\,$using the Python-based FFT ($I({q}_{x},{q}_{y})={\Vert FFT(\rho (x,y){e}^{i{q}_{x}.{u}_{x}})\Vert }^{2}$), we obtain a diffraction pattern (see Fig. 1(e)), which is different from those obtained using the kinematical sum. Indeed, differences are visible in the center of the diffraction peak (inset). In contrary, the FFT of the complex object with a displacement field of opposite sign ($I({q}_{x},{q}_{y})={\Vert FFT(\rho (x,y){e}^{i{q}_{x}.(-{u}_{x})})\Vert }^{2}$ – see Fig. 1(f) gives the same diffraction pattern as those calculated using the kinematical sum. Then, we use the Python-based phasing program to retrieve the complex-valued real-space object. Two solutions arise from phasing: the object and its complex conjugate. It appears that the phasing of the diffraction patterns obtained by the kinematical sum and Fourier transform (with a displacement field of +*u*_*x*_), *i.e*. Figure 1(c,d,f), gives a phase which has indeed an opposite sign compared to the displacement: *ψ* = −*q*_*x*_ . *u*_*x*_. This is explained by the fact that Python uses a negative sign convention for the FFT. The reconstructions obtained by phase retrieval are shown in Fig. 1(g–j) for each case and give the same result as already inferred from diffraction patterns.

For the sake of completeness, we show in Fig. S1 the FFT of the complex object calculated with Mathematica, which uses a positive sign convention. In that case, the FFT of the complex object gives directly the correct diffraction pattern. Most of the phasing algorithms available in the community are either Python or Matlab-based, which use the same negative convention for the FFT. Therefore, a negative sign should be applied to the phase before scaling it back to the displacement. Practically, however, there are only few experimental cases where the correct real-space orientation of the sample is known (*e.g*., when there is an epitaxial relationship between the crystal and the substrate used for crystal growth). In many cases where there is no systematic orientation and asymmetry in crystal shape (*e.g*., batteries^21^), it is not possible to pick up the correct solution arising from the phasing algorithm. Without prior knowledge about the behavior of the displacement (or strain) in the nanocrystal, it is therefore not possible in these cases to discuss the sign of the result. Not knowing the real-space orientation of the crystal is less of a problem when studying defects, because their signature is a jump in the phase^22^, which appears whatever the convention of sign used.

### Simulation model

**Figure 2:**
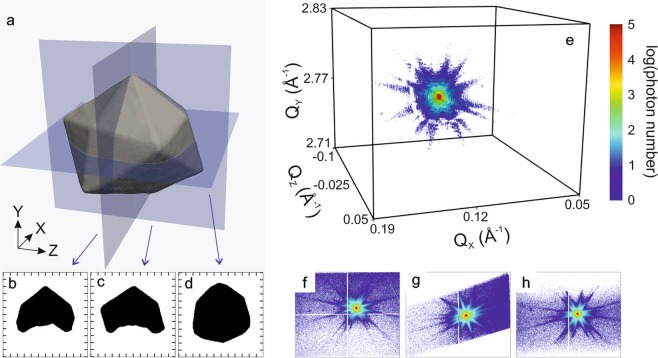
Complex object used for the simulation. (**a**) Isosurface of the support with 2D slices at the center of the array in (**b**) XY plane, (**c**) ZY plane and (**d**) XZ plane. The support is fixed to 1 inside the crystal and 0 outside. (**e**) Contour plot of the experimental 3D diffraction pattern of the THH **111** Pt Bragg peak, where the detector gaps are visible. (**f**–**h**) Diffraction patterns projected onto XY plane, YZ plane and XZ plane, respectively.

To investigate the origin and impact of artefacts on the reconstruction from BCDI measurements, we create a complex-valued real-space object of density $\rho (\overrightarrow{r})$ and phase $\psi (\overrightarrow{r})$: $\tilde{\Omega }(\overrightarrow{r})=\rho (\overrightarrow{r}){e}^{i\psi (\overrightarrow{r})}$. To be realistic, we use the BCDI reconstruction of a real crystal to define the density $\rho (\overrightarrow{r})\,\,$and support (see Fig. 2(a–d)). The support confines the shape and size of the object; it is set equal to *one* inside the crystal and *zero* outside. This crystal is a Pt tetrahexahedral (THH) particle (width of ~400 nm), grown on a glassy carbon substrate by electrochemical deposition and faceting^23^. A scanning electron microscope (SEM) image of the THH particles is shown in Fig. S2. In the following, we use the CXI convention for geometry, *i.e*., Z downstream, Y vertical up and X outboard^24^ (right handed). The Y axis is along the [111] direction of the crystal. For simplicity, we decide to fix the real-space phase, $\psi (\overrightarrow{r})$, constant (flat) equal to zero. Note that the value of the phase itself is not important since after phasing, there is an unknown constant offset in the retrieved phase. We do not simulate noise here, in order to focus our study on reproducible artefacts. We then calculate the diffraction pattern of the complex-valued real-space object $\tilde{\Omega }(\overrightarrow{r})$ using the fast Fourier transform (FFT). The diffracted intensity follows as: ${\rm{I}}(\overrightarrow{q})={\Vert FFT(\tilde{\Omega }(\overrightarrow{r}))\Vert }^{2}$, where $\overrightarrow{q}$ is the scattering vector. The calculated diffraction intensity compares well with the experimental three-dimensional (3D) diffraction pattern of the THH Pt particle displayed in Fig. 1(e), which has been measured around the **111** Pt Bragg peak at the ESRF – the European synchrotron, ID01 beamline^25^. The scattered X-rays were detected using a 2D Maxipix pixel detector (516 × 516 pixels of 55 µm × 55 µm)^26^. The detector is composed of four modules separated by gaps with a size of 220 µm. Pixels at the edge of the module have a size of 165 µm × 55 µm to cover this gap. Depending on the oversampling, the data can be binned and edge pixels used (for an oversampling larger than 3), or masked for smaller oversampling because then it is not possible to redistribute efficiently the measured intensity in virtual pixels of 55 µm × 55 µm. The detector gaps are visible in the contour plot of Fig. 2(e) and in 2D projections in Fig. 2(f–h). In the following, we calculate the 3D diffraction intensity and adjust parameters (detector gaps, dynamic range, *etc*.) to mimic realistic experimental conditions. The generated data are then retrieved using a well-established phase retrieval process to get the real-space object, whose quality is quantitatively evaluated. This allows one to investigate the origin of artefacts in the reconstruction and characterize the performance of BCDI. We do not discuss specifically the phasing algorithm in this work; therefore, all trends presented here apply to the error metric used for phase retrieval (see Methods). The resolution of the reconstruction is estimated using the normalized Phase-Retrieval Transfer Function (PRTF)^27,28^ at a cutoff value of 1/e. The PRTF is a measure of how well the retrieved Fourier amplitudes match the square root of the measured diffraction intensity. Another common method to determine the resolution of experimental data is the Fourier shell correlation^29^.

### Fluctuations in reconstructed data

#### Stripe artefacts

As mentioned in the introduction, it is quite common to observe phase fluctuations in BCDI reconstructions. They often have the form of oscillation stripe artefacts (see, for example refs.^20,28,30^): frequently parallel to facets, they are observed both in the modulus and the phase of the reconstruction and affect the reconstruction statistics. Since the phasing algorithm is based on FFT, it is legitimate to suspect the Gibbs phenomenon and the aliasing effect. The Gibbs phenomenon is known as a non-physical oscillation artefact near the sharp discontinuity^31^, which degrades the quality of the reconstruction. In particular, this phenomenon is noticeable by the cut-off of the waveform in a limited window during the discrete Fourier transform process^32^. Aliasing occurs when the diffraction intensity does not completely decay to zero within the detector area and violates the continuous boundary condition of the Fourier transform^33^. As the phase retrieval algorithms are based on the fast Fourier transform (FFT) algorithm, the reconstructed density and phase are affected by the two effects mentioned above. This is enhanced in the case of a faceted crystal (our case, here): the reciprocal space pattern of a faceted crystal is characterized by streaks of intensity, *i.e*. crystal truncation rods which are normal to the each surface facet. These streaks whose intensity follows a q^−2^ law^34^ can elongate far in reciprocal space and are often truncated by the detector.

Phasing of the experimental data used to generate our model is realized with a FFT window of 270 × 432 × 420 pixels. To avoid as much as possible introducing artefacts in our simulation, we zero-pad the support 3D array to a size of 10^3^ × 10^3^ × 10^3^ pixels before calculating the FFT. Note that in the Bragg geometry, the detector frame is not orthogonal^35^. After phase retrieval, the reconstructed object is interpolated into an orthogonal frame for easier visualization. In order to remain consistent with experiments, we therefore interpolate back the support defined in the orthonormal laboratory frame into the non-orthogonal detector reference frame. Afterwards, the FFT is calculated and the result is normalized to photon counts. The simulated diffraction pattern comprises a total of 5 × 10^7^ photons, which is close to experimental values for this size of transition metal nanocrystal (around 400 nm in diameter). In order to assess the effect of the size of the FFT window, which corresponds to the size of the experimentally exploited detector area, we crop the array, and finally phase it using a Python-based phase-retrieval algorithm, the PyNX package^36^.

**Figure 3:**
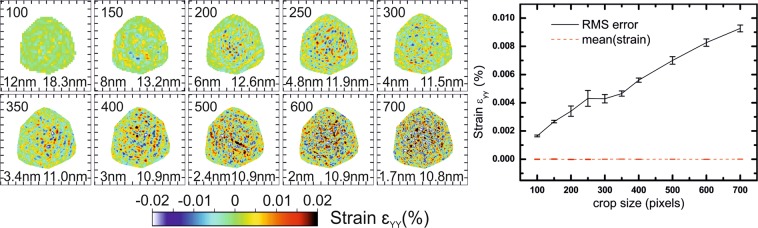
Effect of the cropped size of the FFT window: reconstructions in the XZ plane (Y being the vertical axis). The upper number corresponds to the width of the FFT window in pixels, the bottom left number is the voxel size in nm, and the bottom right number is the resolution obtained from the PRTF. A complex artefact pattern is always present and increases in amplitude when the FFT window is smaller, without affecting strain mean value. Tick spacing corresponds to 50 nm.

The phase obtained from the inversion of the simulated diffraction patterns at the **111** Bragg reflection encodes the displacement field along the [111] direction (u_111_) of the crystal, which is parallel to the Y axis of Fig. 2 and pointing upwards. After removing the non–physical ramp and offset in the retrieved displacement and interpolating back into the laboratory frame, the strain can be derived from the displacement field: ${\varepsilon }_{YY}=\,\frac{\partial {u}_{111}}{\partial Y}$ (Y being vertical). Figure 3 displays slices in the XZ plane through the middle of the reconstructed array of the out-of-plane strain ε_YY_ calculated after phasing. Diffraction patterns for each configuration and slices in XY plane are shown in Figs. S3 and S4 respectively, for different cropped sizes of the FFT window (*i.e*., different sizes of detector). For this particular example, we see that the presence of artefacts is independent of the cropped FFT window size, and that their amplitude increases slowly with the FFT window size. Since we are dealing with a 3D object, stripes interfere with each other, yielding a complex pattern (although a flat phase, *i.e*. no strain, is expected). We use the root-mean-square error (RMSE) of the retrieved strain as an indicator for this trend. The line plot of Fig. 3 shows the RMSE and the mean values of the strain inside the support. The average strain is close to 0 for each cropped size, as expected, while the RMSE reaches 0.009% for a FFT window of 700 × 700 × 700 pixels. A tentative explanation for this trend comes from the fact that we are using a photon counting detector: with larger window size, the number of pixels with intensity rounded to zero photon instead of the non-null Fourier intensity is increasing, therefore the match between the measured and the retrieved diffraction patterns should deteriorate. This is also confirmed by the evolution of the resolution: for small FFT window sizes (100 and to 300 pixels wide), the resolution is affected by the size of the FFT window which cut the data, while from 350 pixels wide and larger FFT window, it is limited by the dynamic range of the noise-free data, *i.e*. the extent of diffracted photons in reciprocal space. If the resolution kept increasing with the window size, it would mean that we could generate reconstructions at any resolution with the same amount of information by just padding the data, which of course is not physical. When the data is not cut by the FFT window, the resolution is determined by the data and not by the detector size. The amplitude of artefacts generated by the FFT window size (*i.e*., the detector area), however, does not seem able to explain by itself all artefacts in reconstructions, which can be experimentally larger by one order of magnitude.

In the following, we decided to fix the detector window to 400 pixels to limit as much as possible aliasing effects, *i.e*., when the intensity does not completely decay to zero within the detector area and violates the continuous boundary condition of the Fourier transform. Aliasing cannot be totally suppressed because of the use of the FFT during iterative phasing: unlike the measurement data, the retrieved complex amplitude array fills totally the FFT window, thus violating the periodic boundary condition.

#### Detector gaps

**Figure 4:**
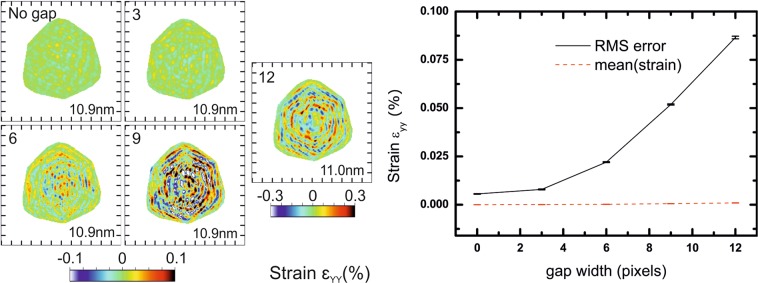
Effect of the presence and width of a gap in the detector: reconstructions in the XZ plane (Y being the vertical axis). The numbers displayed in the top left corner correspond to the width of the gap in pixels, and numbers in the bottom right corner to the resolution obtained from the PRTF. Even for a reasonable gap of 4 to 6 pixels, the RMSE of strain artefacts is larger by an order of magnitude compare to the case without gap. When the gap is larger than 9 pixels, the mean strain value starts to deviate from the null value. Tick spacing corresponds to 50 nm.

The next candidate for potential experimental artefacts is the presence of gaps in the detector (area where no intensity is measured), since they introduce non-random features in the data during the phasing, even if gap voxels are set free during the phase retrieval (the amplitude constraint is not applied on these voxels). Since the 3D diffraction pattern is sampled by successive 2D slices through it, detector gaps are also continuous in the dimension corresponding to the scan. Here, we fix the FFT window size to 400 pixels of 55 µm × 55 µm each and we introduce a gap in the detector. We show the influence of the gap width in Fig. 4 for a gap position arbitrary fixed, with slices in the XZ plane through the middle of the reconstructed array for the ε_YY_ strain. Diffraction patterns for each configuration and slices in XY plane are presented in Figs. S5 and S6, respectively. The RMSE of the strain is obviously very dependent on the presence of a gap and its width. The amplitude of artefacts with realistic gaps (4 to 6 pixels of 55 µm × 55 µm as observed in a Maxipix detector with several modules^26^) is at least one order of magnitude larger than without gaps. The effect of detector gaps is often underestimated, but the above simulations show that this is actually the main contribution to stripe artefacts. Note that the resolution, however, starts to be slightly affected only with gaps larger than 12 pixels. This has a direct implication for experiments using detectors with large gaps (*e.g*., megapixel array detectors): it is necessary to avoid positioning the diffracted/scattered signal in gaps, otherwise it could lead to misinterpretation of the reconstructed data.

**Figure 5:**
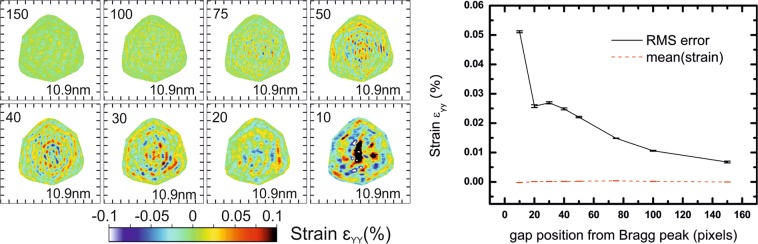
Effect of the distance between gaps and the central Bragg peak position: reconstructions in the XZ plane (Y being the vertical axis). The numbers displayed in the top left corner correspond to the distance of the gap to the Bragg peak in pixels, and numbers in the bottom right corner to the resolution obtained from the PRTF. From this simulation, we conclude that it is good practice to position the gap as far as possible from the diffracted intensity when measuring a BCDI dataset. Tick spacing corresponds to 50 nm.

The position of the gap relative to the Bragg peak is also important. When the crystal has few defects, streaks can go far from the central Bragg peak position in reciprocal space, and the experimentalist may be tempted to put the Bragg peak near the central gap of the detector, in order to collect more signal. In Bragg CDI, there is no redundancy in the data as for ptychography^37^, and missing high-intensity pixels can have a large impact on the algorithm’s ability to reconstruct the crystal. In the following simulation, we fix the FFT window size to 400 pixels of 55 µm × 55 µm, the gap width to 6 pixels, and we vary the distance of the gap to the central Bragg peak position. Figure 5 shows slices in the XZ plane through the middle of the reconstructed array for the ε_YY_ strain calculated after phasing. Diffraction patterns for each configuration and slices in the XY plane are presented in Figs. S7 and S8, respectively. It is clear that the closer the gap is to the Bragg peak, the stronger artefacts it generates. Another indication, that the gap contribution is prominent, is that the spatial frequency of stripe artefacts observed in Fig. 5 is strongly correlated with the distance between the gap and the central Bragg peak position. This level of strain artefact is however too small to have an influence on the resolution.

#### Dynamic range

**Figure 6:**
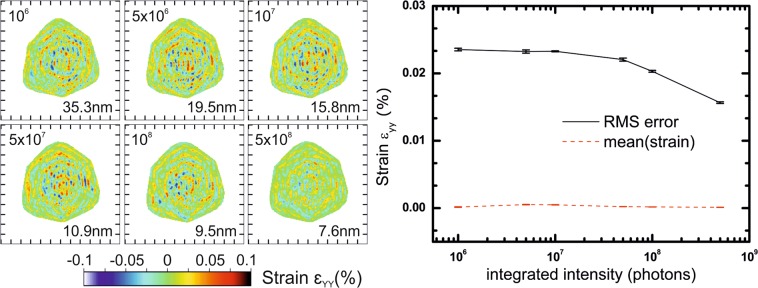
Effect of the dynamic range of the data: reconstructions in the XZ plane (Y being the vertical axis). The number in the top left corner corresponds to the total number of photons in the 3D diffraction pattern, and numbers in the bottom right corner to the resolution obtained from the PRTF. In terms of dynamic range, it is equivalent to 1.2 × 10^4^, 6.2 × 10^4^, 1.2 × 10^5^, 6.2 × 10^5^, 1.2 × 10^6^ and 6.2 × 10^6^, respectively. Although a better dynamic range helps for reducing the amplitude of artefacts, it does not compensate the presence of a gap in the detector. Tick spacing corresponds to 50 nm.

Then, we study the effect of the dynamic range of the data. Keeping the same simulation conditions, *i.e*., a FFT window size of 400 pixels of 55 µm × 55 µm each, a gap width of 6 pixels located 50 pixels away from the Bragg peak, we vary the number of scattered/diffracted photons measured in the detector by renormalizing the diffraction pattern. This is equivalent to tuning the acquisition time of the detector for each 2D slice of the 3D diffraction pattern. Figure 6 shows slices in the XZ plane through the middle of the reconstructed array for the ε_YY_ strain calculated after phasing. Diffraction patterns for each configuration and slices in the XY plane are shown respectively in Figs. S9 and S10. While the voxel size of the reconstruction is related to the FFT window size *via* the formula: (wavelength × sample to detector distance)/(pixel number × pixel size), the resolution of the reconstruction is linked to the dynamic range of the data and the signal to noise ratio. From these noise-free simulations, we see that in otherwise similar conditions, a larger dynamic range slightly reduces the amplitude of artefacts. This effect is however rather limited considering the huge difference in diffracted intensity between various cases.

#### Discussion of simulation results

**Figure 7:**
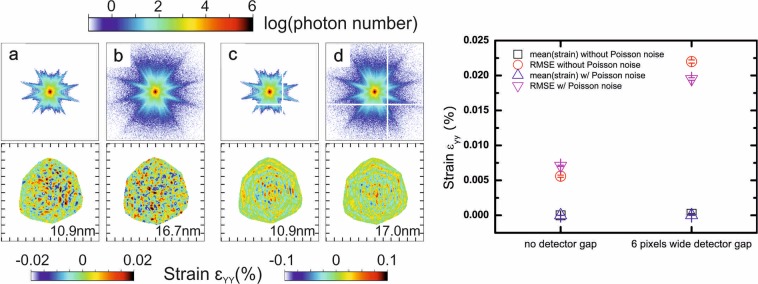
Effect of Poisson noise on previous simulation results. Diffraction patterns and reconstructions in the XZ plane (Y being the vertical axis) for a 400 pixels-wide cropping window and 5 × 10^7^ diffracted photons with: (**a**) no Poisson noise, (**b**) Poisson noise, (**c**) no Poisson noise and a detector gap, (**d**) Poisson noise and a detector gap. Detector gaps are 6 pixels-wide and located 50 pixels away from the Bragg peak. Tick spacing corresponds to 50 nm. Numbers in the bottom right corner correspond to the resolution obtained from the PRTF.

In order to study the impact of various experimental parameters, all simulation results presented above considered a noise-free measurement which is not encountered in experiments. It is legitimate to question whether, in practice, artefacts are not instead dominated by the noise. We present in Fig. 7 the effect of Poisson noise on simulation results for two particular cases. It appears that Poisson noise does not modify the order of magnitude of strain artefacts, but has an impact on the resolution of the reconstruction itself. Therefore, Fig. 7 validates our noise-free approach to understand the origin of strain artefacts.

From the previous simulations, we conclude that the major contribution to stripe artefacts in reconstructions is the presence of a detector gap, its width and distance to the central Bragg peak position. We showed that the size of the FFT window has a smaller impact than detector gaps on strain artefacts, but it can limit the resolution: one wants to enlarge the FFT window in order to avoid cutting intensity streaks. This is equivalent to ensuring a sufficient oversampling in real space: the object should be less than half of the FFT window size^12^. Considering the simulated nanoparticle from this study (Pt nanoparticle of ~400 nm diameter), with an oversampling of ~3 and a resolution limited by the dynamic range, a reasonable gapless detector size would be 400 pixels × 400 pixels of 55 µm × 55 µm each. For 200 angular steps, it corresponds to ~800 Mb of required memory using PyNX for phase retrieval.

There are several perspectives for improving the quality of the reconstruction. We exclude from the present discussion refraction and absorption corrections applied to the output of a phasing algorithm, as they have already been described in the literature^38^. Improvements can be made either by (1) preprocessing the measured data in reciprocal space or (2) post-processing of the outcome of the phasing algorithm. From a conceptual point of view however, it seems more acceptable to work on the reconstructed object than to modify the raw experimental data. Approaches during phasing are not relevant here: genetic algorithm^39,40^ and partial coherence compensation of the incoming X-ray wavefront^41^ do not help to remove stripes since they are just geometric artefacts due to FFT aliasing and appear whatever phasing process is used.

For post-processing, it is common to average independent reconstructions that have enough correlation between them (in the sense of modulus correlation). One drawback of this approach is that the resulting reconstruction may not be anymore the best fit to the diffraction data, in terms of the figure of merit chosen for convergence (the modulus of the average being different from the average of the moduli). Moreover, it results inevitably in a coarsening of the spatial resolution due to tiny differences and slight misalignment between reconstructions (any alignment step implying its own artefacts). Recently, the decomposition of the complex-valued real-space solutions into modes has been introduced^42^. It is similar to the decomposition of modes when reconstructing the probe in ptychography^43^. However, since the most relevant artefacts (as mentioned in this article) are caused by the geometric configuration of the experiment (detector size, presence and position of gaps) and thus part of the experimental data itself, this approach also fails to remove them from the reconstruction. We now approach methods to spatially filter the phase to remove high frequency noise. We introduce first the use of an apodization step after phase retrieval, and then discuss the effect of filtering the reconstructed phase.

#### Apodization

**Figure 8:**
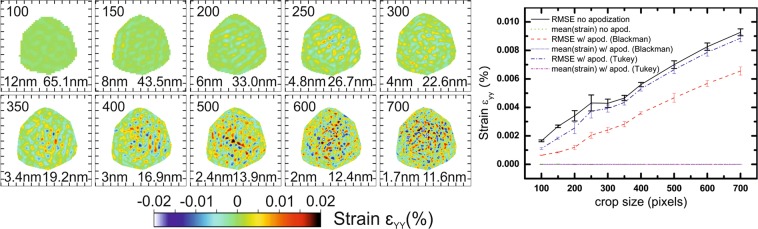
Effect of a single post-phasing apodization step using a Blackman window on the aliasing noise due to the cropped size of the FFT window: reconstructions in the XZ plane (Y being the vertical axis). Only the apodization step has been added to the data processing presented in Fig. 2. The upper number corresponds to the width of the FFT window in pixels, the bottom left number is the voxel size in nm, and the bottom right number is the resolution obtained from the PRTF. Tick spacing corresponds to 50 nm.

Apodization is used commonly in signal processing to compensate discontinuities in the measured spectrum. It smoothly brings a sampled signal down to zero at the edges of the sampled region. For instance, in extended X-ray absorption fine structure (EXAFS), an apodization window is used to avoid spurious mathematical ripples (see for instance, ref.^44^). Various filtering windows exist, but as an example, we choose here to compare two windows, a Blackman window and a Tukey Window of parameter α = 0.7. The shape of these functions is presented in Fig. S11. After phase retrieval, we calculate the diffraction pattern from the reconstructed object, multiply it by the filtering window and calculate back the complex object in real space. The efficiency of this single step at removing the high frequency noise due to the limited size of the FFT window is striking, as shown in Fig. 8. The addition of a single post-phasing apodization step to the overall identical phase retrieval process of Fig. 3 significantly reduced strain noise at small FFT window sizes. It is well known that apodization affects the resolution: there is a tradeoff between cutting high-frequency noise and loss in resolution. The Blackman window, which falls quickly away from its center, has a large impact on the resolution. Tunable windows, like the Tukey window which spans from a rectangular window to a Hann window depending of its parameter α, can be optimized for a particular dataset.

In Fig. S12, we show the effect of apodization on the histogram of modulus distribution: the modulus is more homogeneous, and its mean value has increased. For the sake of completeness, we compare in Fig. S13 post-processing apodization and the application of an apodization to the raw data before phasing. In the latter, the raw data is multiplied by the window function before phase retrieval. A single post-phasing apodization step is more efficient to remove aliasing noise, but causes a larger loss of resolution. Since we are simulating noise-free data, pre-processing has no impact on the data with large FFT windows, where the intensity is already null near the edges of the window.

#### Phase averaging in real space

**Figure 9:**
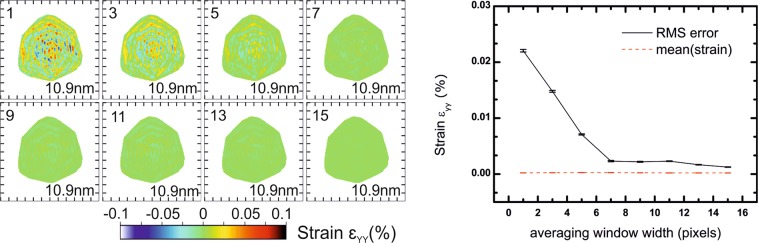
Example of phase averaging: reconstructions in the XZ plane (Y being the vertical axis). The numbers displayed in the top left corner correspond to the width in pixels of the 3D averaging window, and numbers in the bottom right corner to the resolution obtained from the PRTF. There is a significant decrease of the RMSE value of strain by one order of magnitude when the averaging window size is similar or larger than the spatial frequency of stripe artefacts. Averaging the phase is a valid approach for this dataset where no high frequency variation is expected in the phase. Tick spacing corresponds to 50 nm.

In the following, we show the effect of averaging the phase of the reconstruction using a 3D window. It allows to filter spatially the phase and to remove high frequency features that are supposedly not physical. Averaging is realized sequentially in each frame corresponding to different rocking angles, since the resolution may be different from dimensions corresponding to the detector plane. Keeping the same simulation conditions, *i.e*. a FFT window size of 400 pixels of 55 µm × 55 µm each, a gap width of 6 pixels located 50 pixels away from the central Bragg peak, we vary the size of the averaging 3D window. Near the surface, only pixels belonging both to the support and the averaging window are taken into account. Figure 9 displays slices in the XZ plane through the middle of the reconstructed array for the ε_YY_ strain calculated after phasing. Slices in the XY plane are shown in Fig. S14.

The resolution here is not affected by averaging the phase, which can be explained by the flat phase model used for these simulations. Phase averaging may, however, hide special features in the object like phase shifts due to defects. Near the surface layer, voxels outside the support cannot be used for averaging (the phase being random there), hence the average has to be done with less voxels, and is more prone to statistical noise. Averaging the phase can therefore be used only for a qualitative approach, when no feature with a spatial frequency larger than artefacts is expected in the phase. In order to be closer to experimental cases, we compare the effect of phase averaging and apodization on real experimental data in Fig. S15, where the nanocrystal has a defect. It is clear from Fig. S15 that phase averaging is not an option in the presence of defects.

#### Impact of isosurface on the evaluation of surface strain

**Figure 10:**
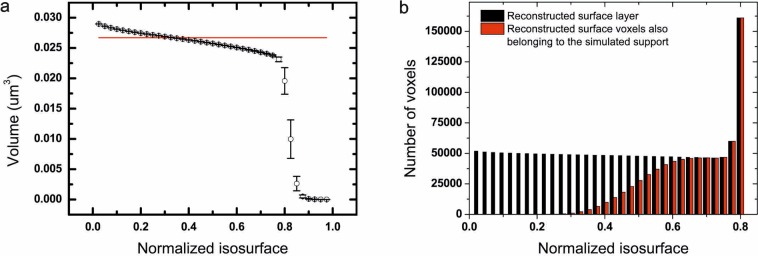
(**a**) Reconstructed support volume depending on the isosurface. The red line corresponds to the volume of the model support. (**b**) Number of voxels in the reconstructed surface layer (in black) which also belong to the model strain surface layer (in red).

**Figure 11:**
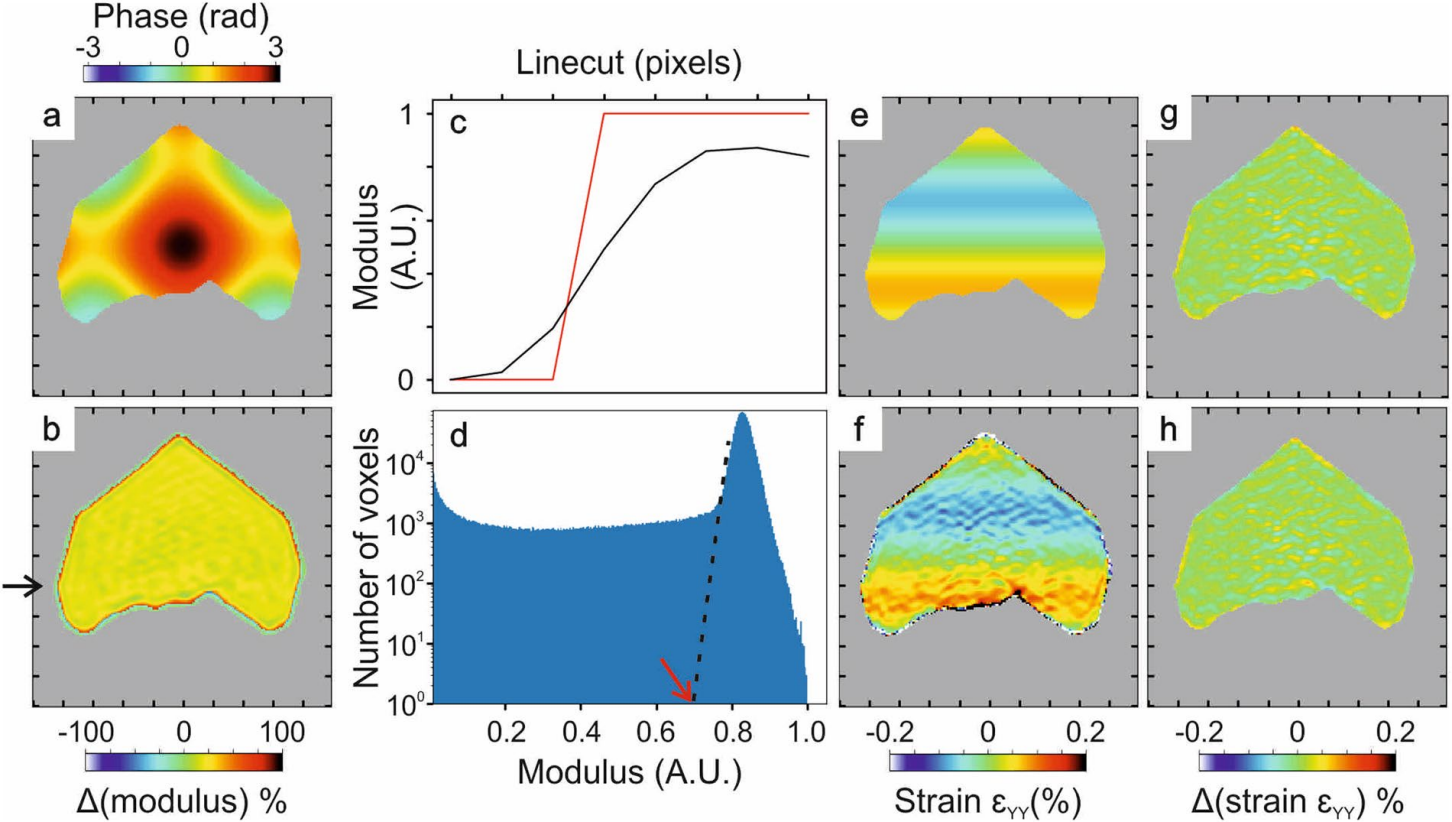
(**a**) Phase model used for the simulation: slice in the XY plane (Y being the vertical axis). (**b**) Difference of the simulated modulus and the retrieved modulus. (**c**) Line cut through an edge (at the arrow position in (**b**)) of simulated and phased modulus. (**d**) Histogram of the complete 3D modulus distribution and empirical criterion for isosurface level determination. (**e**) Simulated and (**f**) retrieved strain ε_YY_. (**g**) Difference between the simulated strain (red) and the retrieved strain (black), with a too low isosurface (32.5%) corresponding to volume conservation. (**h**) Difference between the simulated strain and the retrieved strain, with an isosurface determined by our criterion (70%). Tick spacing corresponds to 50 nm. The background has been artificially set to grey outside the support.

In a number of cases, the quantity of interest is the strain at the surface of the nanocrystal. The surface in BCDI corresponds to the surface voxel layer defined by a modulus isosurface. Indeed, the normalized modulus ranges from 0 to 1, but there is no criterion yet on how one should define the proper isosurface to extract the proper strain values. Moreover, for a sharp interface, the strain will be defined only from the top sublayer to the core, because the gradient of the phase at the surface is calculated between a point on the crystal and a point outside. BCDI reconstructions from real data show a smoother interface because the crystal shape is convoluted with the resolution function, which is also direction dependent^28^. To assess these questions, we use the same model as previously but with a known phase, which is shown in Fig. 11(a). Due to anisotropic displacement, the diffraction pattern is no more centrosymmetric (Fig. S15). We first present the dependence of the reconstructed volume on the isosurface in Fig. 10(a), where the red line represents the volume of the model support. Regarding the conservation of the volume, the correct isosurface is ~32.5%. However, since the strain is not defined at the surface voxel layer, a higher isosurface has to been used to visualize it. In Fig. 10(b), we show the number of voxels in the reconstructed surface layer which also belong to the strain surface layer of the model. The two numbers start to match at ~70%, which we define at the correct isosurface for surface strain visualization. Starting from ~75%, the number of voxels in the reconstructed starts to increase: the ‘surface’ being defined by the isosurface on the modulus, it is the manifestation of holes starting to appear in the support for too high isosurfaces (holes surface layer being added to the surface by this method).

The phase pattern used for the simulation gives rise to a strain ε_YY_ of the order of 0.1% maximum as shown in Fig. 11(e). The difference between the simulated modulus and the reconstructed modulus is presented in Fig. 11(b), with a line cut through an edge in Fig. 11(c). As explained above, the limited resolution of the reconstruction leads to smoother interfaces compared to the model: the edge of the reconstructed modulus (black curve) is not as sharp as the simulated one (red curve. In Fig. 11(d), we present our empirical criterion for isosurface determination: the value of the isosurface level is taken at the foot of the peak of the histogram of the complete 3D modulus distribution. Note that taking a larger isosurface results in holes in the reconstructed modulus. In this noise-free simulation, the vertical axis of the modulus histogram is in logarithmic scale, but for experimental data, a linear scale would be more appropriate. If we plot the reconstructed strain for voxels where the modulus is non zero (Fig. 11(f)), we clearly see at the surface that few layers present strain values that are not physical, only due to the gradient of the phase with voxels not belonging to the support, convoluted with the resolution function. The correct isosurface is the one for which those unphysical surface layers are removed. In Fig. 11(g), we show the difference between the simulated and the retrieved strain ε_YY_ for a too low isosurface level (32.5%, which corresponds to the volume conservation of the support in Fig. 10(a)), versus an isosurface determined using our criterion (~70%) in Fig. 11(h).

While 2D slices in Fig. 11(g,h) look similar (slight differences appearing only at the surface voxel layer), the effect of choosing a wrong isosurface is obvious in Fig. S17, where we show the 3D isosurface representation of the simulated and retrieved crystals using the various post (pre)-processing filtering methods presented in this study. Isolating the surface layer using the coordination number of voxels in the support, we can study the deviation of the reconstructed strain compared to the simulated one depending on the data process. We summarize in Table 1 the RMSE for the surface voxel layer and the bulk (where the surface voxel layer has been removed). 3D phase averaging yields the best result in terms of discrepancy between the simulated strain and the retrieved strain. Considering that the maximum absolute strain in the model is 0.0756%, the error is still substantial in the retrieved strain at the surface voxel. Note that in this work, to remain general, we do not tailor the phase retrieval algorithm to fit at best this particular simulation model, since it does not affect the overall discussion about isosurface determination.

**Table 1:** RMSE strain values obtained for the surface voxel layer and the bulk of the nanocrystal, depending on the data processing scheme.

	No phase average (32.5%)	No phase average (70%)	Apodization before phasing (70%)	Apodization after phasing (70%)	3D phase average over 7 pixels (70%)
RMSE at the surface voxel layer (%)	(56.8 ± 2.1) × 10^−3^	(37.3 ± 0.8) × 10^−3^	(34.6 ± 0.3) × 10^−3^	(31.3 ± 0.5) × 10^−3^	(24.6 ± 0.4) × 10^−3^
RMSE in the bulk (%)	(18.0 ± 0.2) × 10^−3^	(14.1 ± 0.3) × 10^−3^	(11.8 ± 0.09) × 10^−3^	(10.3 ± 0.2) × 10^−3^	(8.29 ± 0.05) × 10^−3^

Apodization was realized using a Blackman window. Values in parentheses correspond to the isosurface used for the statistical analysis.

## Conclusion

We present in this article a detailed study of artefacts in BCDI reconstructions induced by both dataset collection and phase retrieval algorithms (*i.e*., for instance, aliasing due to detector size and detector gaps, detector dynamic range, *etc*.). The methodology used in the paper helps to isolate statistical errors from systematic errors that are inherent in experimental setups. The resolution in the retrieved strain is limited by artefacts which can reach 10^−3^ when large masked areas are present in the dataset. The best approach is, when possible, to avoid using a detector with gaps. Regarding the size of the FFT window used for phasing, the best practice is to select the smallest window containing signal, while not truncating the diffraction pattern in order to avoid any unnecessary loss in resolution. Finally, adding a post-processing filtering step can help further reducing high-frequency noise. BCDI is a technique applied increasingly to *in situ* or *operando* systems, in which the initial qualitative approach tends to be replaced by a quantitative analysis of displacement and strain for probing subtle changes under real application conditions. We provide two important milestones for this, namely the relation between the retrieved phase and displacement field depending on the FFT sign convention of the phasing algorithm, and an empirical criterion for isosurface determination. We believe that this study will contribute to improving the reliability of the BCDI technique towards a quantitative strain analysis method.

## Methods

### Sample preparation

Pt THH particles were synthesized on glassy carbon (GC) electrodes by a square-wave-potential method with a saturated calomel reference electrode (SCE) and a Pt foil counter electrode in 2 mM H_2_PtCl_6_ and 0.1 M H_2_SO_4_ electrolyte following the procedure described in ref.^23^. The GC electrode is first subjected to a potential of +1.20 V for 2 s to clean the surface and then −0.35 V for 60 ms to create Pt nuclei. The Pt nuclei grows into THH particles by applying a square-wave-potential between +0.04 V and +1.09 V at 100 Hz for 10 min.

### Experimental setup details

BCDI measurements were performed at the upgraded ID01 beamline^25^ of the ESRF synchrotron. The required beam size was obtained with a Kirkpatrick-Baez mirror, which focused the beam down to ≈300 nm (horizontal) × 165 nm (vertical) full width at half maximum, as determined by the ptychographic reconstruction of the probe using a Siemens star. The sample was positioned out of the focus of the beam to ensure the full illumination of a single nanoparticle by the beam. A coherent portion of the beam was selected with high precision slits by matching their horizontal and vertical gaps with the transverse coherence lengths of the beamline: 200 μm (vertically) and 60 μm (horizontally). The intensity distribution around the **111** Pt reflections was measured in a vertical coplanar diffraction geometry. The BCDI experiment was performed at a beam energy of 9 keV. The diffracted beam was recorded with a 2D MAXIPIX^26^ photon-counting detector positioned on the detector arm at a distance of 0.5 m. The detector distance as well as the rocking angle increment were chosen in order to ensure oversampling of interference fringes (oversampling ~3). A typical counting time was 1 s per angle, to get good resolution while preserving the stability of the particle. The sample was mounted on a Physik Instrumente Mars xyz piezoelectric stage with a lateral stroke of 100 μm and a resolution of 2 nm, sitting on a hexapod that was mounted on a (3 + 2 circles) goniometer.

### Simulation details

3D simulations were realized using the same pipeline as for experimental data. Using a support retrieved from a real dataset, a complex object was created using a particular phase pattern (flat or not). Then, the data was interpolated back into the detector plane using the same geometric parameters as the experiment. The diffraction pattern was calculated on a 10^3^ × 10^3^ × 10^3^ grid to avoid as much as possible introducing aliasing and converted into photons to a defined total diffracted intensity. No noise was introduced into the simulated diffraction pattern. Then, phase retrieval was performed as described in the next section.

For 2D simulations, we used an asymmetric 2D core-shell model with a particular phase. The diffraction pattern was then calculated using FFT or a kinematic sum. Finally, phase retrieval was performed as described in the next section.

### Phase retrieval

Phase retrieval was carried out on the raw diffracted intensity data using PyNX package^36^, imposing at each iteration that the calculated Fourier intensity of the guessed object agrees with the measured data. The metric used to estimate the goodness of fit during phasing was the free log-likelihood^42^, available in PyNX. Defective pixels for experimental data and gaps in the detector were not used for imposing the reciprocal space constraint mentioned above and thus were evolving freely during phasing. The initial support, which is the constraint in real space, was estimated from the autocorrelation of the diffraction intensity. A series of 1400 Relaxed Averaged Alternating Reflections (RAAR^45^) plus 200 Error-Reduction (ER^46,47^) steps, including shrink wrap algorithm^48^ every 20 iterations were used.

For 3D simulations and experimental data, we kept only the best 5 reconstructions from 200 with random phase start and a known support. For experimental data, which was measured in a focused beam out of focal plane, the phasing process included a partial coherence algorithm to account for the partially incoherent incoming wave front^41^. The reconstruction was then corrected for refraction and absorption, the small size of the particles ensuring that dynamical diffraction effects could be neglected^38^. After removing the phase ramp and phase offset, the data was finally interpolated onto an orthogonal grid for easier visualization. For each simulated case, we calculated the resolution (PRTF) and error bars for strain based on these five independent reconstructions. We used a single reconstruction to make figures.

For 2D simulations, convergence of the phasing algorithm was more challenging because of the complex electron density and phase configuration of the model. To ensure the best reconstruction possible, we kept only the best 50 reconstructions from 1000 with random phase start and a known support (800 RAAR + 200 ER), and performed the decomposition in modes^42^.

### Apodization

We applied apodization either before or after phase retrieval. Before phase retrieval, it consists of multiplying the 3D raw data array by the 3D apodization window, which was chosen as a Blackman window or a Tukey window of parameter α = 0.7.

For post-processing apodization, the Fourier transform of the reconstructed object was calculated in order to go back to the frequency domain. Then, we multiplied it by the 3D filtering window of the same size as the reconstructed array. Finally, we calculated back the complex object in real space by applying a FFT.

## Supplementary information

supplementary information

## Data Availability

The data reported in this paper is available upon request. The simulation scripts belong to the BCDI package (10.5281/zenodo.3257617), that can be downloaded from PyPI (https://pypi.org/project/bcdi/) or GitHub (https://github.com/carnisj/bcdi). The phasing algorithm PyNX is available at http://ftp.esrf.fr/pub/scisoft/PyNX/.
