# Supplementary material for: Towards a quantitative determination of strain in Bragg Coherent X-ray Diffraction Imaging: artefacts and sign convention in reconstructions
Source: Sci Rep. 2019 Nov 22;9:17357. doi: 10.1038/s41598-019-53774-2 (PMC6874548; doi:10.1038/s41598-019-53774-2)
Supplement: Supplementary file 1 — supplementary information [file 41598_2019_53774_MOESM1_ESM.pdf]

Towards a quantitative study of strain in Bragg Coherent X-ray Diffraction Imaging: artefacts and sign convention in reconstructions.

Jérôme Carnis[1,2,*], Lu Gao[3], Stéphane Labat[1], Young Yong Kim[4], Jan Philipp Hofmann[3], Steven J. Leake[2], Tobias U. Schülli[2], Emiel J. M. Hensen[3], Olivier Thomas[1] & Marie-Ingrid Richard[1,2].

[1]Aix Marseille Université, CNRS, Université de Toulon, IM2NP UMR 7334, 13397, Marseille, France.
[2]ID01/ESRF, The European Synchrotron, 71 Avenue des Martyrs, 38000 Grenoble, France.
[3]Laboratory for Inorganic Materials and Catalysis, Department of Chemical Engineering and Chemistry, P. O. Box 513, 5600 MB Eindhoven, The Netherlands.
[4]Deutsches Elektronen-Synchrotron (DESY), D-22607 Hamburg, Germany.
*jerome.carnis@desy.de

Supplementary Materials

Justification of the use of Fast Fourier Transform for phase retrieval:

For a crystal, the scattering amplitude of the X-rays in the kinematical approximation verifies[1]:

$$A(\boldsymbol{q}) = \sum_{n=1}^{N} F_n(\boldsymbol{q}) e^{-i\boldsymbol{q}.\boldsymbol{u}(\boldsymbol{R_n})} e^{-i\boldsymbol{q}.\boldsymbol{R_n}}$$

where $\overrightarrow{\boldsymbol{R}_n}$ is the position of the nth unit cell in the perfect lattice and $\overrightarrow{u_j}$ its displacement from the perfect lattice.

with $F_n(\boldsymbol{q})$ the structure amplitude of the nth cell:

$$F_n(\boldsymbol{q}) = \sum_{j=1}^{S} f_{nj}(\boldsymbol{q}) e^{-i\boldsymbol{q}.\boldsymbol{r}_j}$$

and $f_{nj}(\boldsymbol{q})$ the atomic scattering factor of an atom j in the unit cell n:

$$f_{nj}(\boldsymbol{q}) = \int \rho_{nj}(\boldsymbol{r}') e^{-i\boldsymbol{q}.\boldsymbol{r}'} d\boldsymbol{r}'$$

We assume that the structure factors of the different cells are identical ($F_n(\boldsymbol{q}) = F(\boldsymbol{q})$).

Assuming that $\|(\boldsymbol{q} - \boldsymbol{h}) \cdot \boldsymbol{u}(\boldsymbol{R_n})\| \ll 2\pi$ (where $\boldsymbol{h}$ is the scattering vector at the position of the Bragg reflection), we can approximate $\boldsymbol{q} \cdot \boldsymbol{u}(\boldsymbol{R_n})$ by $\boldsymbol{h} \cdot \boldsymbol{u}(\boldsymbol{R_n})$ and we obtain the Fourier transform:

$$A(\boldsymbol{q}) = F(\boldsymbol{q}) \sum_{n=1}^{N} e^{-i\boldsymbol{q}.\boldsymbol{u}(\boldsymbol{R_n})} e^{-i\boldsymbol{q}.\boldsymbol{R_n}} \approx F(\boldsymbol{q}) FT(e^{-i\boldsymbol{h}.\boldsymbol{u}(\boldsymbol{R_n})})$$

This last approximation is generally used for the analysis of coherent Bragg imaging experiments as it allows a fast Fourier transform computation.

Supplementary reference:

[1] I. A. Vartanyants & I. K. Robinson. Partial coherence effects on the imaging of small crystals using coherent x-ray diffraction. *J. Phys.: Condens. Matter* 13, 10593–10611 (2001).

Supplementary figures (Fig. S1-S17):

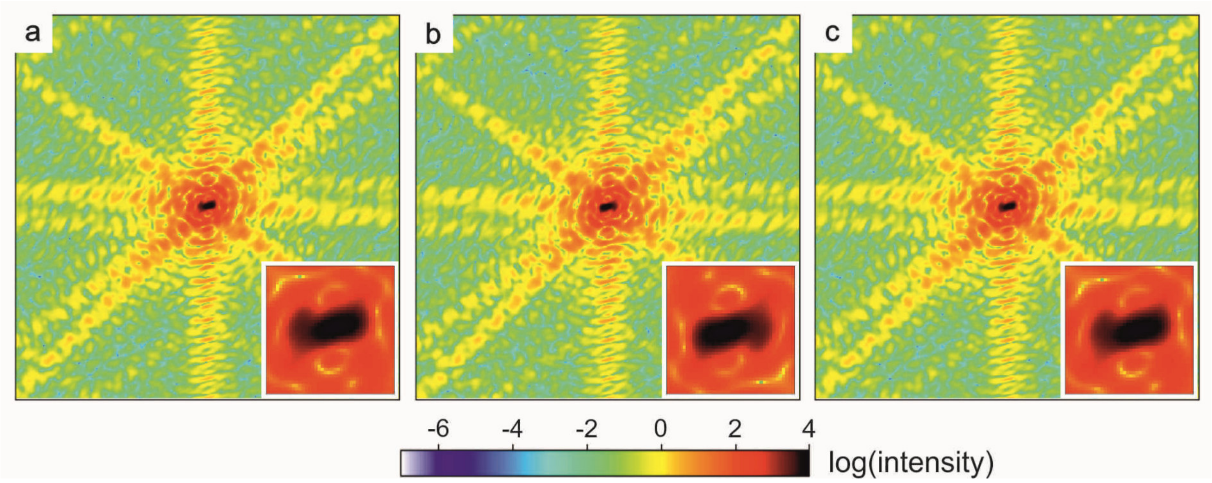

Fig. S1 : Comparison of diffraction patterns calculated with FFT symmetric normalization: a) using a positive FFT convention (example here with Mathematica), b) using a negative FFT convention (example here with Python) and c) using a negative FFT convention (Python) and the FFT of a complex object with displacement field of opposite sign ($-u_x$). The inset in all the figures displays the center of the calculated Bragg reflection. There is no phase flip for FFT calculated with the positive convention like that of Mathematica.

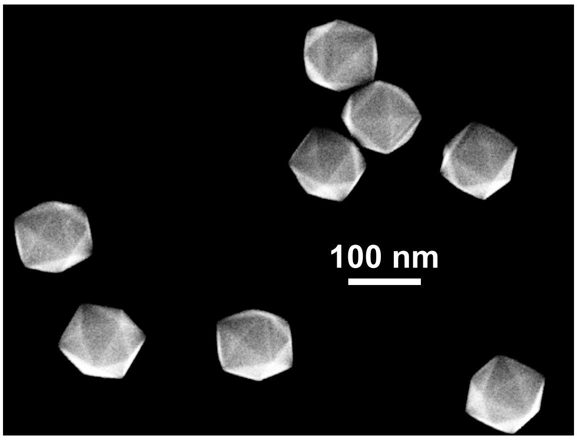

Fig. S2: Scanning electron microscope (SEM) image of tetrahexahedral (THH) Pt particles on glassy carbon.

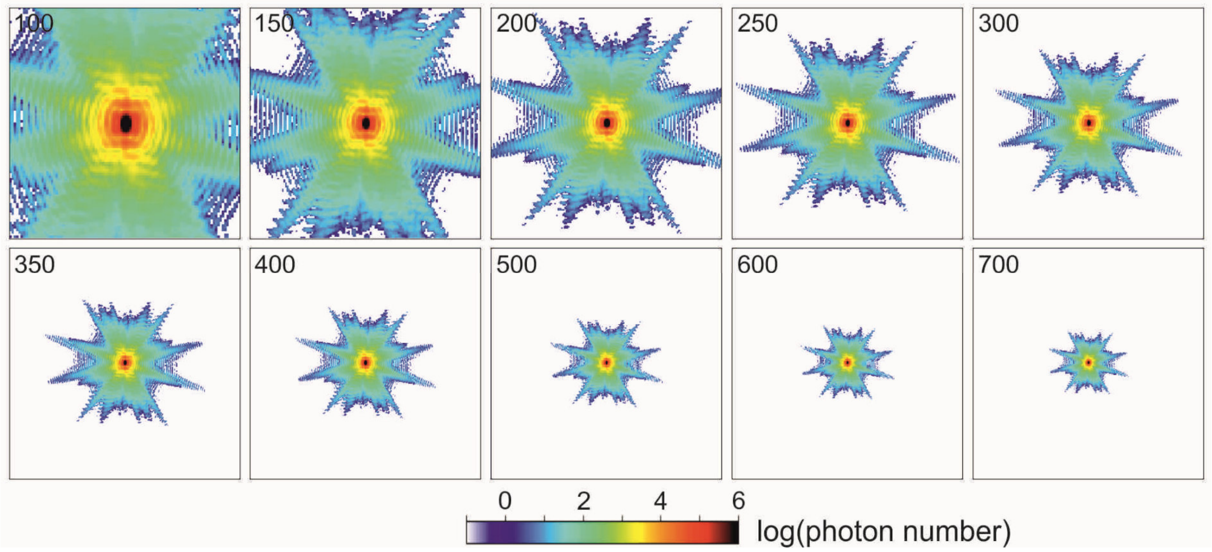

Fig. S3 Sum perpendicular to the detector plane of 3D diffraction patterns of the same integrated intensity (5x10^{7}photons in total in the original array of 10^{3}x10^{3}x10^3 pixels) cropped to different sizes (see number at the top-left corner for each image), before phase retrieval. There are no gaps in the detector.

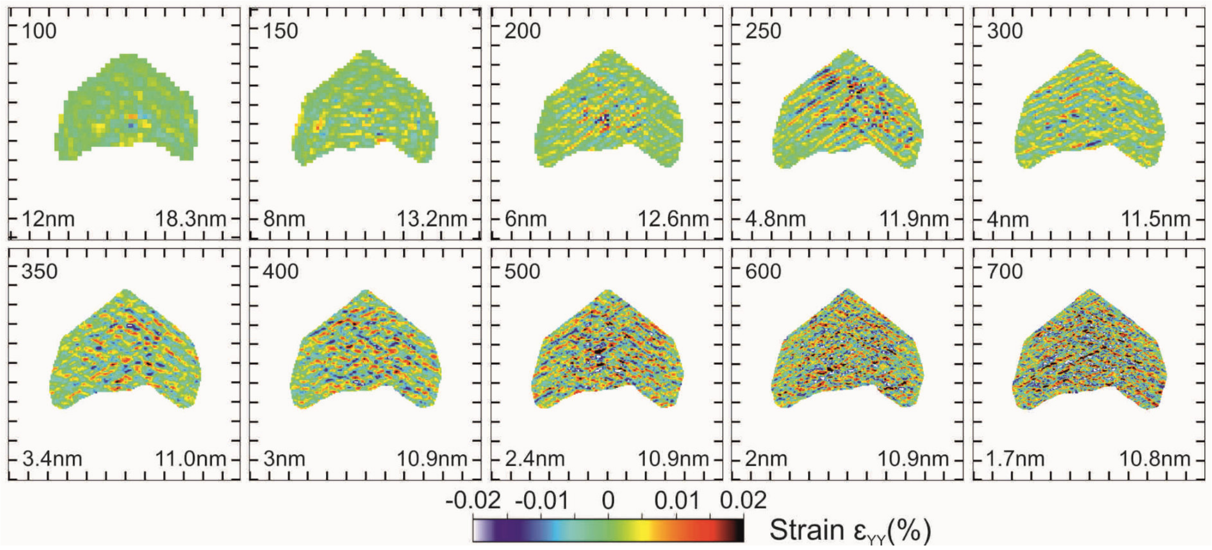

Fig. S4: XY (Y being the vertical axis) central slice of the reconstructed out-of-plane strain (ε_{yy}) for diffraction patterns presented in Fig. S2. The distance between two ticks corresponds to 50 nm. The number at the top left corner corresponds to the width of the FFT window, the number at the bottom left corner to the voxel size in real space and the number at the bottom right corner to the resolution obtained from the PRTF.

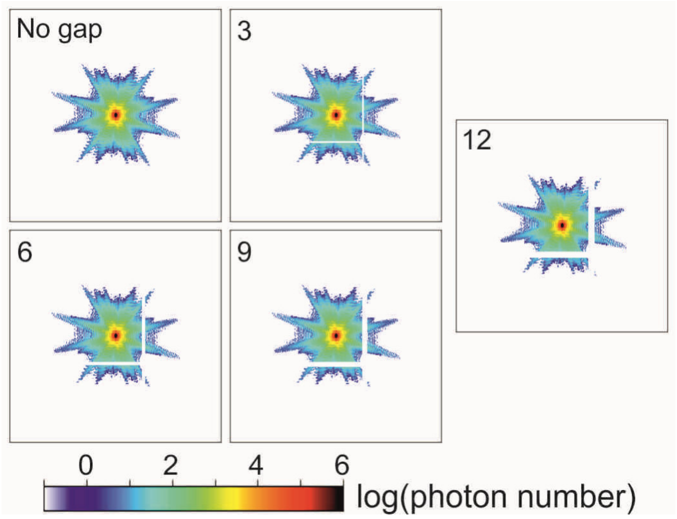

Fig. S5: Model diffraction patterns for the study of gap width influence on strain artifacts. Numbers correspond to the gap width in pixels. The FFT window width is fixed to 400 pixels, the gap is located at 50 pixels away from the Bragg peak in each dimension of the detector plane, and the total number of photons is fixed to 5×10^{7}. The number at the top left corner corresponds to the gap width in pixels.

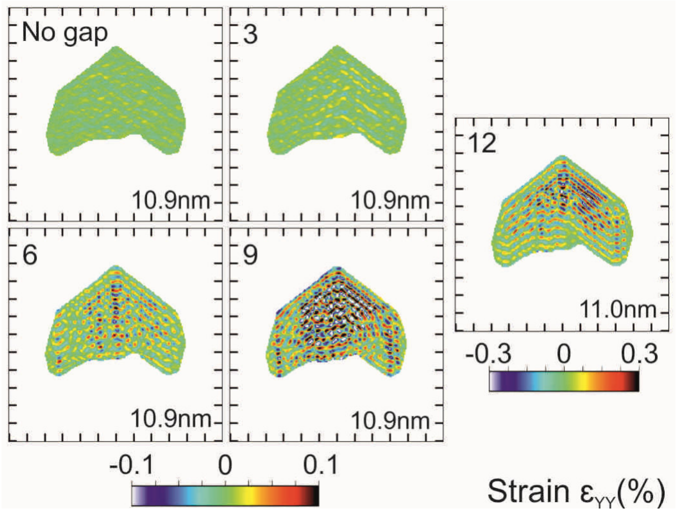

Fig. S6: XY (Y being the vertical axis) central slice of the reconstructed out-of-plane strain (ε_{yy}) for diffraction patterns presented in Fig. S4. The distance between two ticks corresponds to 50 nm. The number at the top left corner corresponds to the gap width in pixels, and the number at the bottom right corner to the resolution obtained from the PRTF.

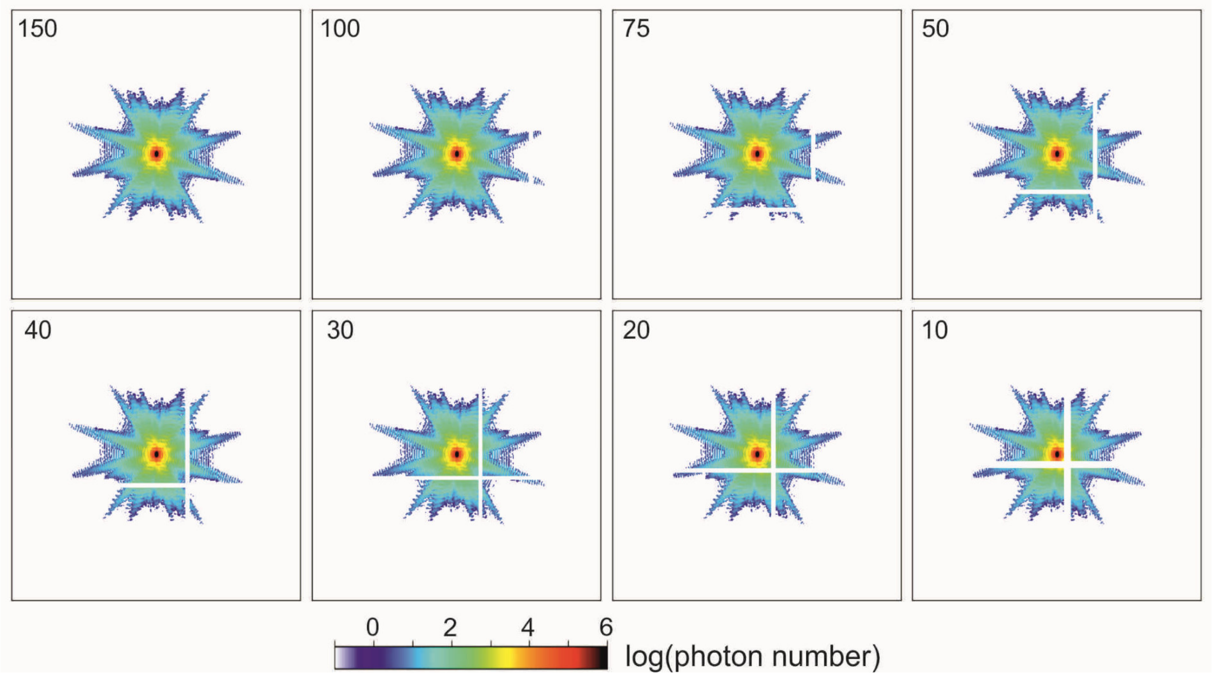

Fig. S7: Model diffraction patterns for the study of gap position influence on strain artifacts. Numbers correspond to the distance of the gap to the Bragg peak in pixels. The FFT window width is fixed to 400 pixels, the gap width is fixed to 6 pixels in each dimension of the detector plane, and the total number of photons is fixed to 5×10^7 photons.

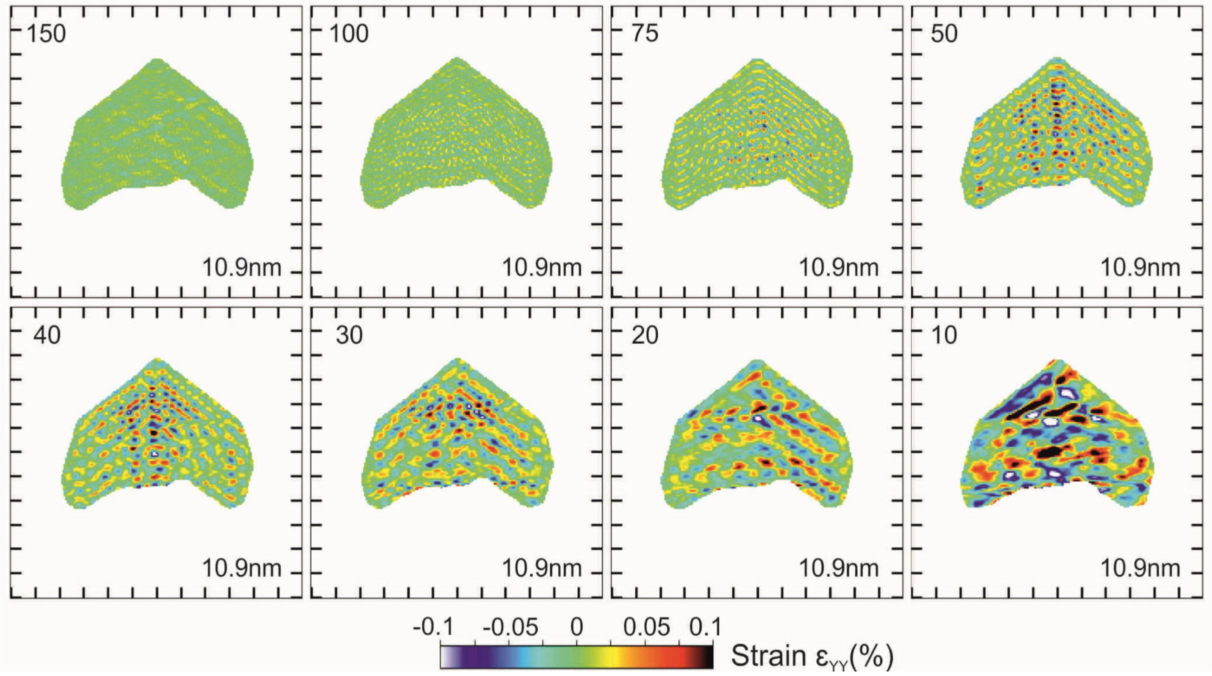

Fig. S8: XY (Y being the vertical axis) middle slice of the reconstructed out-of-plane strain (ε_{yy}) for diffraction patterns presented in Fig. S6. The distance between two ticks corresponds to 50 nm. The number at the top left corner corresponds to the distance of the gap to the Bragg peak in pixels, and the number at the bottom right corner to the resolution obtained from the PRTF.

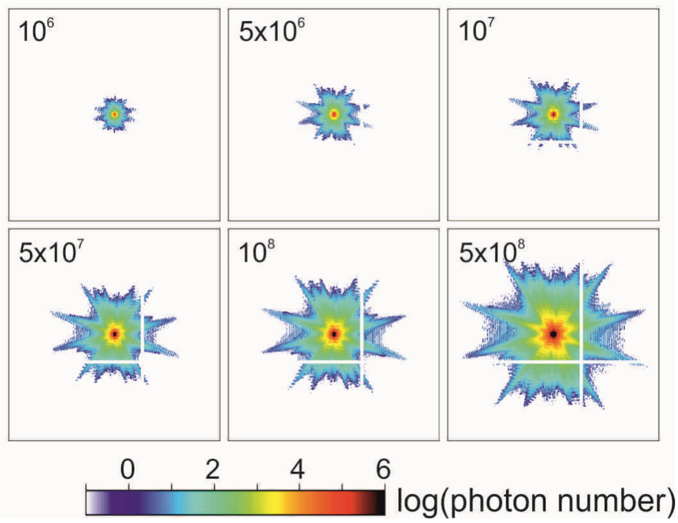

Fig. S9: Model diffraction patterns for the study of the influence of dynamic range on strain artifacts. Numbers correspond to the total integrated intensity in photons in the diffraction pattern. In terms of dynamic range, it is equivalent to $1.2x10^4$, $6.2x10^4$, $1.2x10^5$, $6.2x10^5$, $1.2x10^6$ and $6.2x10^6$ respectively. The FFT window width is fixed to 400 pixels, the gap width is fixed to 6 pixels and the gap is positioned 50 pixels away from the Bragg peak in each dimension of the detector plane.

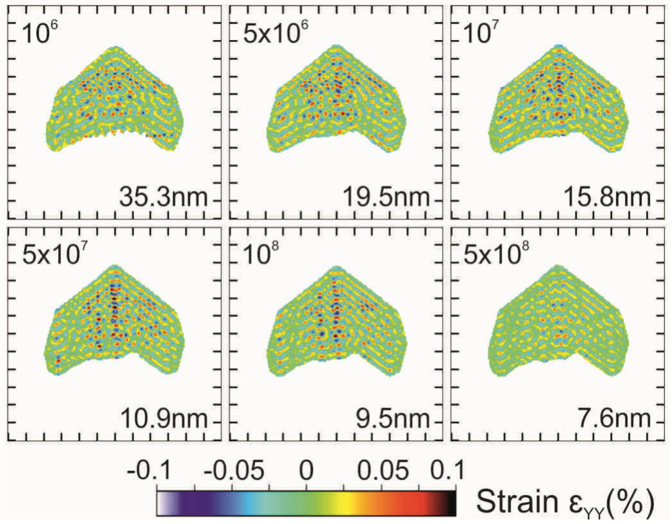

Fig. S10 : XY (Y being the vertical axis) middle slice of the reconstructed out-of-plane strain (ε_{yy}) for diffraction patterns presented in Fig. S8. The distance between two ticks corresponds to 50 nm. Numbers at the top left corner correspond to the total integrated intensity in photons in the diffraction pattern. In terms of dynamic range, it is equivalent to $1.2x10^4$, $6.2x10^4$, $1.2x10^5$, $6.2x10^5$, $1.2x10^6$ and $6.2x10^6$ respectively. The number at the bottom right corner corresponds to the resolution obtained from the PRTF.

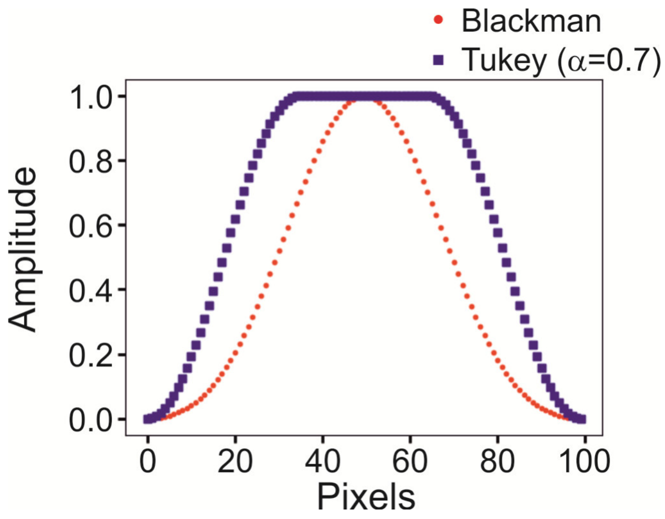

Fig. S11: Window functions used in this study for apodization. The Tukey window spans from a rectangular window when α=0 to a Hann window when α=1.

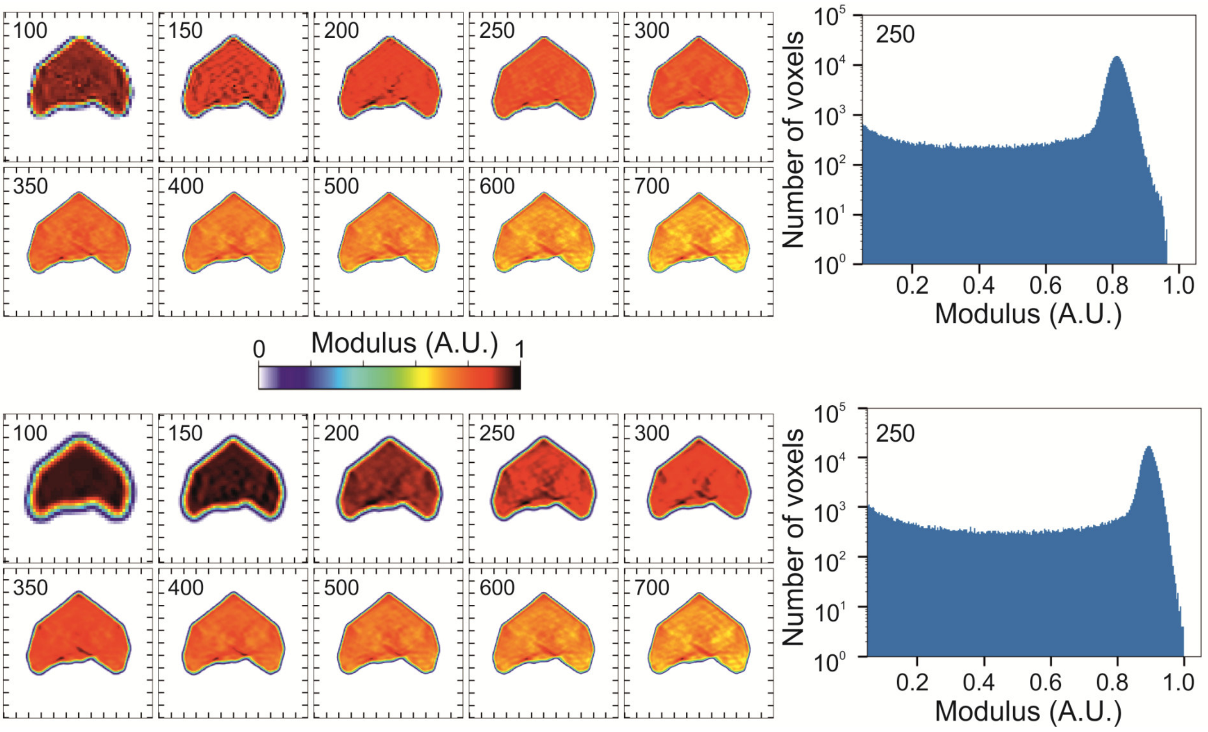

Fig S12: Effect of post-phasing apodization on the modulus. The top two rows are the retrieved modulus without apodization, and the lower two rows show the corresponding modulus with a single post-phasing apodization step using a Blackman window. The effect on the histogram of modulus distribution for a particular FFT window size is shown on the right. The number corresponds to the FFT window size.

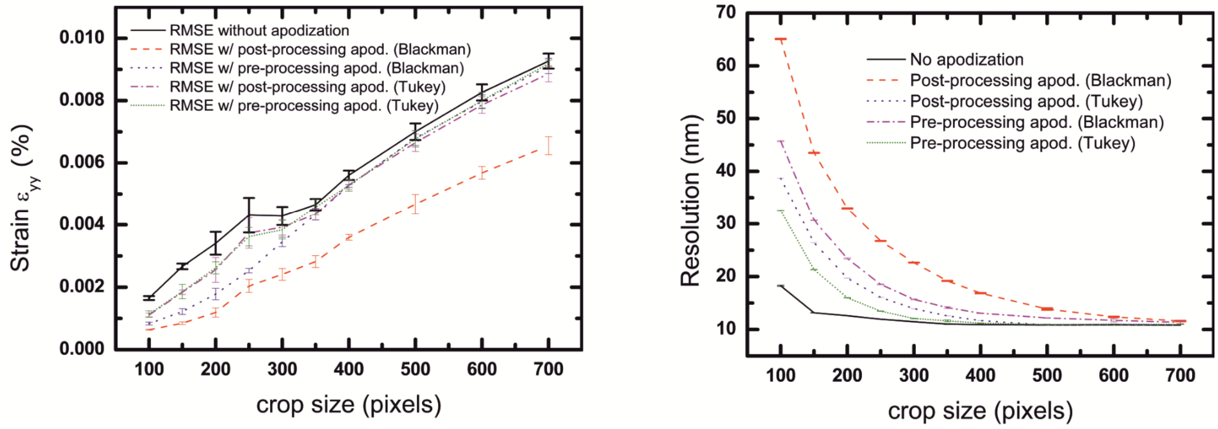

Fig S13: (Left) Comparison of the RMS error of the retrieved strain obtained for different cropping sizes of the FFT window. The model used has a phase equal to zero inside the support. Apodization involves multiplying the data in reciprocal space by a filtering window. For pre-processing apodization, the measured diffracted intensity is multiplied by the filtering window before phasing, while for post-processing apodization, the FFT of the reconstructed complex object is multiplied by the filtering window and then Fourier transformed back. (Right) Resolutions obtained from the PRTF for different cropping sizes of the FFT window.

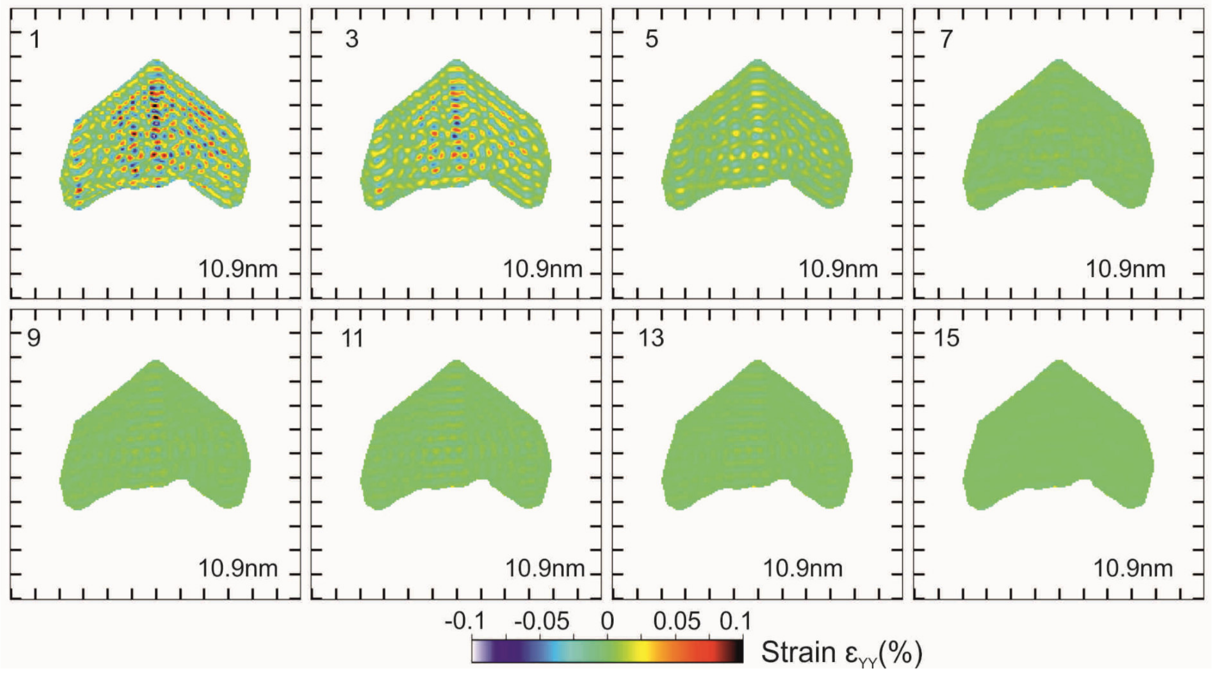

Fig. S14: XY (Y being the vertical axis) central slice of the reconstructed out-of-plane strain (ε_{yy}) for various widths of the 3D phase averaging window. The FFT window width is fixed to 400 pixels, the gap width is fixed to 6 pixels in each dimension of the detector plane and the gap is positioned 50 pixels away from the Bragg peak in each dimension of the detector plane. The corresponding diffraction pattern is the one with an integrated intensity of $5x10^7$ in Fig. S8. The distance between two ticks corresponds to 50 nm. The number at the top left corner corresponds to the width of the 3D phase averaging window and the number at the bottom right corner to the resolution obtained from the PRTF.

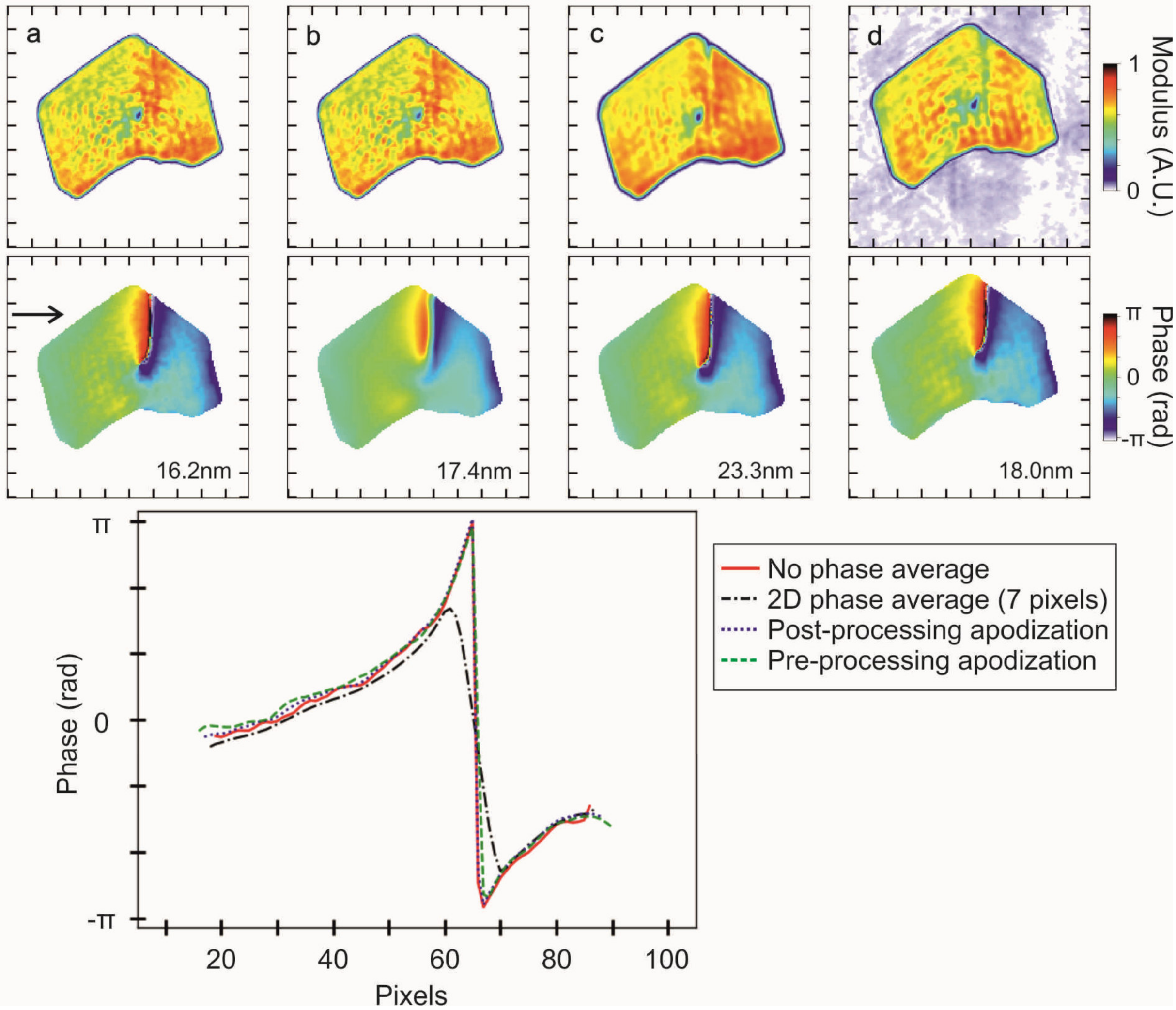

Fig. S15: Effect of averaging the phase reconstructed from an experimental dataset, in a presence of a defect (*i.e.* 2π phase jump). a) Modulus without averaging. b) Modulus when phase is averaged over a 7x7 pixels wide 3D window. c) Modulus with a single post-phasing apodization step (Blackman window). d) Modulus with pre-processing apodization (Blackman window). The corresponding phases are shown below the modulus. The space between ticks corresponds to 50nm. The number in bottom right corner corresponds to the resolution obtained from the PRTF. In e), we show line cuts through the defect, extracted at the position of the arrow. The pixel size is 3nm.

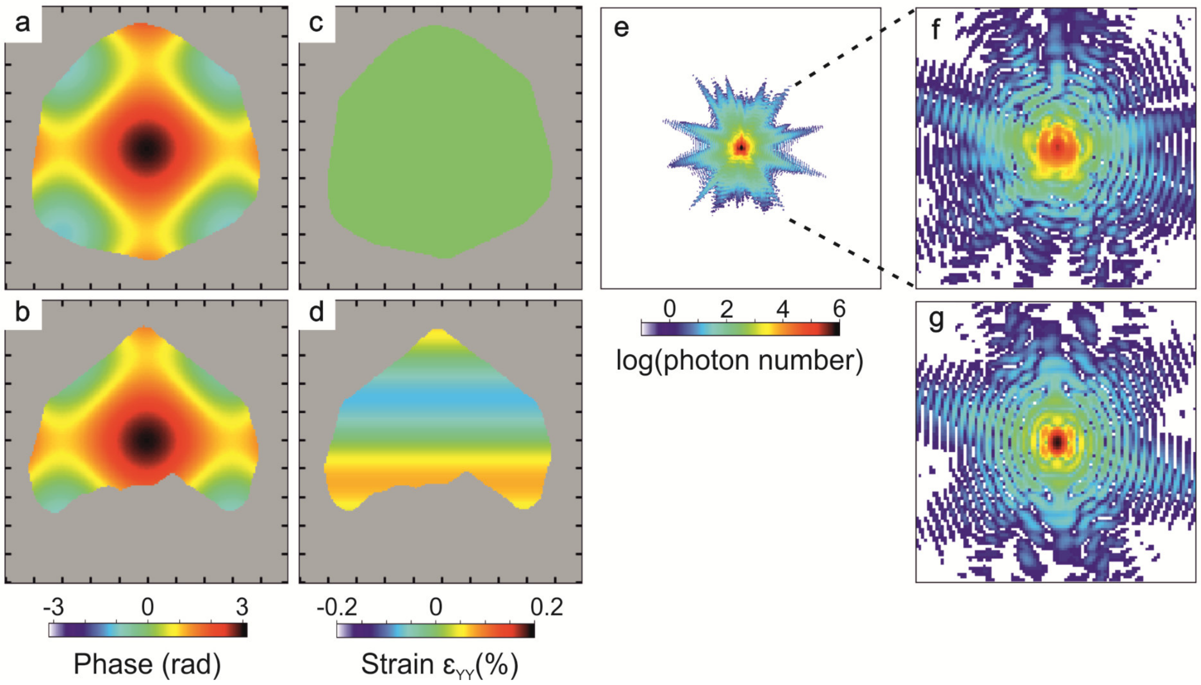

Fig. S16: Phase model used for the study of isosurface influence on surface strain. a) Slice in the XZ plane and b) in the XY plane (Y being vertical). c) and d) Corresponding strain ε_{YY} in the respective planes. e) Diffraction pattern of the model, summed along Z. f) Zoom to the center of the Bragg peak (middle slice in Z) for the model and g) for the bare support without strain. The diffraction pattern is no longer centro-symmetric in presence of strain. The background has been artificially set to grey outside the support.

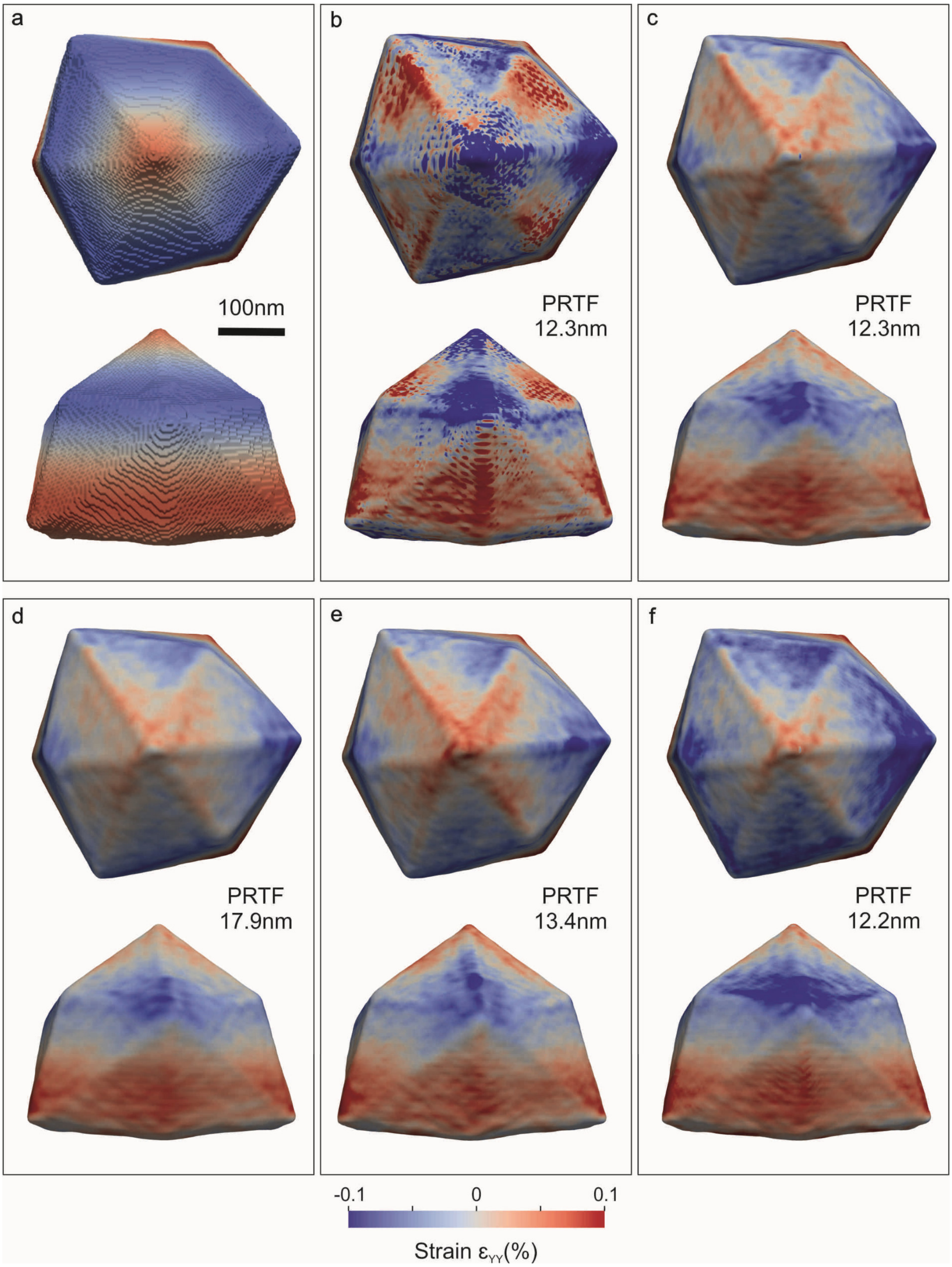

Fig. S17: Comparison between simulated and retrieved strain ε_{YY}. a) Simulated strain plotted on the support in XZ plane (top) and XY plane (bottom). b) Retrieved strain plotted on a too low isosurface (32.5%) corresponding to the volume conservation. Using our criterion (70%) the retrieved strain plotted on an isosurface (c); (d) with a single post-processing apodization step, (e) with a pre-processing apodization step using a Blackman window. (f) retrieved strain calculated from the phase averaged over 7 pixels. We also indicate the resolution obtained from the PRTF.
